# Neighborhood-Level Poverty at Menarche and Prepregnancy Obesity in African-American Women

**DOI:** 10.1155/2016/4769121

**Published:** 2016-06-22

**Authors:** Andrea E. Cassidy-Bushrow, Rosalind M. Peters, Charlotte Burmeister, Lawrence F. Bielak, Dayna A. Johnson

**Affiliations:** ^1^Department of Public Health Sciences, Henry Ford Hospital, One Ford Place, Detroit, MI 48202, USA; ^2^College of Nursing, Wayne State University, 5557 Cass Avenue, Detroit, MI 48202, USA; ^3^Department of Epidemiology, University of Michigan, 1415 Washington Heights, Ann Arbor, MI 48109, USA; ^4^Division of Sleep and Circadian Disorders, Brigham and Women's Hospital and Harvard Medical School, 221 Longwood Avenue, Boston, MA 02115, USA

## Abstract

*Introduction*. Menarche is a critical time point in a woman's reproductive system development; exposures at menarche may influence maternal health. Living in a poorer neighborhood is associated with adult obesity; however, little is known if neighborhood factors at menarche are associated with prepregnancy obesity.* Methods*. We examined the association of neighborhood-level poverty at menarche with prepregnancy body mass index category in 144 pregnant African-American women. Address at menarche was geocoded to census tract (closest to year of menarche); neighborhood-level poverty was defined as the proportion of residents living under the federal poverty level. Cumulative logistic regression was used to examine the association of neighborhood-level poverty at menarche, in quartiles, with categorical prepregnancy BMI.* Results*. Before pregnancy, 59 (41%) women were obese. Compared to women in the lowest neighborhood-level poverty quartile, women in the highest quartile had 2.9 [1.2, 6.9] times higher odds of prepregnancy obesity; this was slightly attenuated after adjusting for age, marital status, education, and parity (odds ratio: 2.3 [0.9, 6.3]).* Conclusions*. Living in a higher poverty neighborhood at menarche is associated with prepregnancy obesity in African-American women. Future studies are needed to better understand the role of exposures in menarche on health in pregnancy.

## 1. Introduction

Prepregnancy obesity is associated with adverse pregnancy and birth outcomes, including increased risk of gestational diabetes, preeclampsia, caesarean section, and having a large-for-gestational-age neonate [1, 2]. Prepregnancy obesity rates in the US are high, affecting approximately 1 in 5 pregnancies [3]. African-American women have increased risk of prepregnancy obesity [4]. There is growing interest in intervening on weight during the preconception phase to improve perinatal outcomes [5]. Importantly, intervening earlier, such as during puberty, may have a greater impact on preventing or reducing the burden of obesity in pregnancy as child and adolescent obesity is strongly associated with adulthood obesity [6].

There is limited, but growing, evidence regarding the role of childhood factors and experiences with obesity during pregnancy [7, 8]. Menarche is a critical time point in a woman's sexual development, reflecting significant biological changes and signaling the transition to the reproductive phase [9]. Obesity in childhood is associated with early age of menarche [10]. The association between obesity at menarche and adult obesity, however, is not fully explained by childhood obesity nor with age at menarche [11, 12]. Age at menarche may be a proxy for sexual maturation reflecting the influence of other factors, such as social and economic conditions, that affect biological maturity [12].

In developed countries such as the US, poverty increases risk of obesity, particularly in women; it is hypothesized that this association may be due to factors such as limited access to high quality, inexpensive food, or perceived discrimination over the life course [13]. Neighborhood-level poverty at the census tract level is one contextual factor that can be used to capture socioeconomic status (SES) over time and in a variety of populations including in children who have little control over their own socioeconomic standing [14]. While neighborhood-level poverty (measured in childhood) is associated with obesity in the transition from adolescence to young adulthood [15] and currently living in a poor area is associated with obesity in pregnancy [16], to our knowledge, no studies have examined associations of neighborhood-level poverty at time of menarche with prepregnancy obesity. A life-course approach to studying obesity that focuses on different developmental stages and exposures during those stages [17] may be especially relevant in the study of obesity in nonwhite groups [18]. Thus, we examined if neighborhood-level poverty at the time of menarche was associated with prepregnancy obesity in a sample of pregnant, African-American women.

## 2. Methods

### 2.1. Sample

The study population and data collection procedures have been described in detail elsewhere [19, 20]. A total of 203 pregnant African-American women in their 2nd trimester being seen for prenatal care at Henry Ford Health System in metropolitan Detroit, MI, were recruited for a study visit. Women provided written informed consent and the study was approved by the Institutional Review Boards at the participating institutions.

### 2.2. Prepregnancy BMI

As part of routine prenatal care, women were asked to self-report their height and weight from the time just before they became pregnant. Prepregnancy body mass index (BMI, in kg/m^2^) was calculated. Prepregnancy obesity was defined as BMI ≥ 30, overweight as BMI ≥ 25 and <30, normal weight as BMI > 18.5 and <25, and underweight as BMI ≤ 18.5; only 8 women were classified as underweight and thus were combined with the normal weight category for analysis. Consistent with other studies [21], self-reported prepregnancy BMI was highly correlated (*r* = 0.97; *P* < 0.001) with the 1st measured BMI during pregnancy obtained from the prenatal medical record (measured at mean 9.4 ± 3.7 weeks of gestation).

### 2.3. Menarche and Neighborhood-Level Poverty

Age at menarche was self-reported and time since menarche calculated as the difference between age at study visit and age at menarche. Women were asked to think about when they got their first period and then they were asked to report the address where they were living at the time of menarche. If women were unable to report an address, they were asked if they could report what the major cross streets were where they lived. A few participants reported intersections (*n* = 7), and those were used to identify the closest postal address. We used participant self-reported year of menarche to identify the appropriate census year to obtain neighborhood-level poverty. One participant began menarche in the late 1970s and her address was mapped to the 1980 census tract; participants reporting menarche between 1980 and 1985 were also mapped to 1980 census tracts. Those who reported menarche between 1986 and 1995 were mapped to 1990 census tracts. Finally, we mapped participants reporting menarche in 1996 and later to 2000 census tracts. Since census tract boundaries may change over time, we used the geocoded address at menarche to obtain data for the appropriate census tract corresponding to the year of census as described above. A similar approach to defining poverty during puberty in a study of premenopausal women was used elsewhere [22].

Neighborhood-level poverty was defined as the proportion of residents in a census tract living below the federal poverty level (using poverty level at the time of the corresponding census). In our sample, at menarche, participants lived in neighborhoods that ranged from having 3.4% of residents living under the federal poverty level to having 63.1% of residents living under the federal poverty level. Mooney et al. recently demonstrated that estimates of the association of neighborhood contextual factors, based on census level variables, on health outcomes may result in biased estimates if the continuously distributed factor is used [23]. However, use of quantiles to partition the contextual factor results in unbiased estimates [23]; thus, we calculated sample quartiles of neighborhood-level poverty at the time of menarche. In our sample at menarche, quartile 1 consisted of women living in neighborhoods where the proportion of residents under the federal poverty level ranged from 3.4 to 14%; Quartile 2 ranged from 14 to 29%; Quartile 3 ranged from 29 to 38%; and quartile 4 consisted of women living in neighborhoods where at least 38% of residents lived under the federal poverty level.

Women also reported current residential address. We utilized a similar approach to obtain current neighborhood-level poverty. Addresses were mapped to the US Census Bureau's American Community Survey 2006–2010 data to obtain census tract and the corresponding census tract poverty level.

Further, in order to assess individual-level SES at the time of menarche, participants were asked to report the maximum level of education of their mother; to differentiate this from the participant's education, this is referred to as* grandmother's* education.

### 2.4. Covariates

Women self-reported race, marital status, and household income. Parity (number of previous viable pregnancies) was obtained from the maternal medical record.

### 2.5. Statistical Analysis

All analyses were conducted using SAS 9.4. Participant characteristics were compared by prepregnancy BMI category using chi-square or Fisher's exact test for categorical variables and ANOVA or Kruskal-Wallis for continuous variables. Cumulative logistic regression was used to examine the association of neighborhood-level poverty at menarche in quartiles with categorical prepregnancy BMI. Models were fit unadjusted (model I), adjusted for maternal age, marital status, and maternal education at the time of pregnancy (model II), and finally additionally adjusted for parity (model III).

We conducted several sensitivity analyses. First, childhood SES is often associated with adulthood SES. Models II and III were fit additionally adjusted for current neighborhood-level poverty quartiles. Second, individual-level SES at the time of menarche may be a potential confounding variable. A previous study found maternal education to be more strongly associated with obesity than paternal education [24]; thus, we utilized* grandmother's* education as our measure of individual-level childhood SES. Several women (*n* = 8) had missing data for* grandmother's* education; to preserve sample size for the sensitivity analysis, mean imputation was used and models II and III were fit additionally adjusted for* grandmother's* education. Finally, since longer time since menarche may be associated with greater risk for obesity, we refit our final models adjusting for time since menarche instead of maternal age.

## 3. Results

As described previously [19], 3 women were* a priori* excluded due to prepregnancy morbid obesity preventing accurate assessment of weight with standard scales. Five women missing age at first period and five women reporting an address at menarche outside of Michigan were excluded. Sixteen women reported a complete address at menarche that was not mappable to the appropriate census tract and 30 women reported insufficient address information at menarche to allow for geocoding. Our final analytic sample consisted of 144 (72%) women able to report a valid residence at menarche. There was minimal clustering by neighborhood with the 144 women residing in 131 neighborhoods at the time of menarche.

There were no significant differences in women with and without reported address at menarche with respect to prepregnancy BMI (*P* = 0.52), age at menarche (*P* = 0.25), or age at study visit (*P* = 0.91).

### 3.1. Prepregnancy BMI

Overall, mean prepregnancy BMI of women was 29.0 ± 7.4 kg/m^2^; 59 (41.0%) women were obese at the start of pregnancy. Participant characteristics are presented by prepregnancy BMI category in Table 1. Time since menarche, age at study visit during pregnancy, marital status, parity, and being nulliparous were each statistically significantly associated with prepregnancy BMI category (all *P* < 0.05). Although obese participants had slightly younger age at menarche than overweight and normal weight participants, this was not statistically significant.

### 3.2. Neighborhood Poverty

At menarche, participants were living in neighborhoods where, on average, 27 ± 13% of residents were living below federal poverty levels, which is consistent with US estimates of childhood poverty between 1980 and 2000 [25]. Neighborhood-level poverty at menarche was statistically significantly and positively associated with parity (*P* = 0.005) and inversely associated with being nulliparous (*P* = 0.004) but was not statistically significantly associated with any other descriptive factor (all *P* > 0.1; data not shown).

### 3.3. Relationship between Poverty and BMI

Mean neighborhood-level poverty at menarche was higher among women in higher prepregnancy BMI categories (Table 1; *P* = 0.054).  Table 2 presents the association of neighborhood-level poverty at menarche, in quartiles, with prepregnancy BMI category. In unadjusted models, women in the highest compared to lowest quartiles of neighborhood-level poverty at menarche had statistically significantly increased odds of being in a higher BMI category (*P* = 0.049). After adjusting for maternal age at study visit, maternal education, and marital status, compared to women in the lowest quartile of neighborhood-level poverty at menarche, women in the fourth quartile of poverty had increased odds of being in a higher prepregnancy BMI category compared to women in the 1st quartile (*P* = 0.048). After further adjusting for parity, the association was slightly attenuated and was no longer statistically significant (*P* = 0.09).

### 3.4. Sensitivity Analysis

There was a statistically significant and positive correlation between neighborhood-level poverty at menarche and current neighborhood-level poverty (*r* = 0.19, *P* = 0.021). In a model adjusted for maternal age, marital status, maternal education, and current neighborhood-level poverty, neighborhood-level poverty at menarche remained statistically significantly associated with prepregnancy obesity (*P* = 0.048); women in the highest compared to lowest quartile of neighborhood-level poverty at menarche had statistically significantly increased odds (OR = 2.7; 95% CI 1.0, 7.1) of being in a higher BMI category. This was slightly attenuated after further adjusting for parity (OR = 2.5; 95% CI 0.9, 7.0; *P* = 0.078). In a model adjusting for maternal age, maternal education, marital status, and* grandmother's* education [i.e., measure of childhood SES], the association between neighborhood-level poverty and prepregnancy BMI category was slightly attenuated (OR = 2.6; 95% CI 1.0, 6.8; *P* = 0.057). Further adjusting for parity, the association between neighborhood-level poverty and prepregnancy BMI category was slightly attenuated (OR = 2.4; 95% CI 0.9, 6.6; *P* = 0.092). Finally, in a model adjusting for time since menarche, marital status, and maternal education, the association of neighborhood-level poverty was slightly attenuated (OR = 2.3; 95% CI: 0.9, 5.9; *P* = 0.096) and remained elevated; however it was nonsignificant after adjusting for parity (OR = 2.1; 95% CI: 0.8, 5.7; *P* = 0.145).

## 4. Discussion

We provide first-time, observational evidence suggesting that living in a higher poverty neighborhood at the time of menarche is associated with greater prepregnancy obesity risk in African-American women. The association between neighborhood-level poverty at menarche and prepregnancy obesity remained elevated, although it was no longer statistically significant, after adjusting for parity. Our findings are consistent with a recent study showing lower neighborhood SES at puberty, but not in earlier childhood, was associated with lower levels of sex hormone binding globulin in a sample of 143 premenopausal, nonpregnant women (mean age 36.8 ± 5.5 years; 32.2% African-American) [22]; sex hormone binding globulin levels increase during pregnancy, with lower levels associated with measures of obesity [26].

Neighborhood poverty may impact obesity in several ways [27]. Poorer neighborhoods tend to have lower quality food retailers (i.e., more convenience and liquor stores and/or fast food restaurants) which may promote, by necessity, poorer eating habits [28]. This is often coupled with limited access to safe avenues for physical activity [29]. African-American women in particular may adopt unhealthy behaviors in early life, especially overeating of “comfort foods,” as a learned, coping strategy to manage chronic stress [30]. Together, along with increased exposure to crime and other stressors, these factors may be contributing to prepregnancy obesity. Such exposures may be especially relevant during puberty [31]. In mouse models, stress (shipping) specifically experienced during puberty results in altered behavioral response to hormones and changes in the hypothalamic-pituitary adrenal (HPA) axis response [32]; dysregulation of the HPA axis activity is associated with obesity [33] and thus provides a potential biologic mechanism linking neighborhood poverty at menarche with prepregnancy obesity. Whether this is mediated through HPA axis activity, however, would require future study.

In our sample of African-American women, 41.0% were obese prepregnancy; this is higher than prepregnancy obesity rates in African-American women (31.5%) from 9 states in the Pregnancy Risk Assessment Monitoring System (PRAMS) [4]. In contrast, only 20.5% of Caucasian women were obese before pregnancy in PRAMS [4]. Data from Michigan, where obesity rates are nearly 37% among African-American adults [34], was not included in PRAMS which may explain why obesity rates are higher in our study.

In a recent study, the disparity in obesity rates between African-American and Caucasian youth was explained by neighborhood economic deprivation [27]. Neighborhood-level factors in adolescence, such as poverty, could directly promote disparities in prepregnancy obesity seen in adult women in the US. Our analysis focused on neighborhood at the time of menarche; although this is a critical stage in reproductive development [9] and thus may have particular relevance for health during pregnancy, future studies should capture other early-life time points (e.g., neighborhood at birth, at age 18 years) to establish and test the life-course approach.

After adjusting for parity, the association between neighborhood-level poverty and prepregnancy obesity was attenuated and no longer statistically significant. Higher poverty is associated with higher pregnancy rates [35]. Higher parity is also associated with increased prepregnancy obesity risk [4], as weight from previous pregnancies is often maintained into subsequent pregnancies. Because parity may be an intermediate variable in the association of neighborhood-level poverty at menarche with prepregnancy BMI, adjusting for it in our final models may have led to an overadjustment bias [36]. Similarly, the association between neighborhood poverty at menarche and obesity was attenuated when we adjusted for time since menarche instead of maternal age. Earlier age at menarche is associated with adult obesity [12] as well as with growing up in poverty [37] and thus future studies are needed to determine whether earlier age at menarche may act as a confounder or intermediate variable in the association of neighborhood poverty at menarche with prepregnancy obesity.

In the US, over half (51%) of pregnancies are unintended [38], making preconception programs to reduce the burden of maternal obesity during pregnancy challenging. Further, black women in the US have the highest rate of unintended pregnancy (69% of pregnancies) [38]. A recent Cochrane systematic review concluded that there were no randomized controlled trials that evaluated the impact of preconception interventions in overweight and obesity [39]. If the risk for prepregnancy obesity originates during puberty, the ideal time to intervene may be during adolescence. However, during pediatric well-child visits, pubertal topics are addressed less frequently than recommended [40] and may represent a gap in care that could improve future maternal and child health.

There are several limitations. Given the observational nature of the study, we cannot provide causal evidence of a relationship between neighborhood poverty at menarche and prepregnancy obesity. The association between neighborhood-level poverty at menarche and prepregnancy obesity, although only slightly attenuated, was not robust to inclusion of parity. Future studies with a larger sample size are needed to better understanding if parity is a confounding factor or a mediating variable. A number of women were unable to report address at menarche; while we found no differences between women who were and were not able to report address at menarche in selected characteristics, our results may be biased by this missing data. As done elsewhere [8], our primary outcome variable was based on self-reported prepregnancy weight. Although there was high correlation between first measured pregnancy weight and self-reported prepregnancy weight in our study and others have shown that categorization of prepregnancy weight comparing self-report to first measured pregnancy weight is similar [41], our results may still be subject to self-report bias. We did not have measures of body size at menarche, so we were unable to account for potential mediating effects via menarche body size. We assigned neighborhood poverty to women based on the decennial census year closest to year of menarche; this may not fully represent the neighborhood condition at menarche and may have introduced increased variability in the exposure assessment. While women's ability to recall age at menarche has been shown to be valid [42], the ability to recall residence at menarche has not been explored; thus we may be subject to recall bias. However, because menarche is a central, unique event of puberty, memory of characteristics surrounding this event (i.e., “flashbulb memories”) may have enhanced recall of residential location at this time [42]. Any misclassification due to reporting errors in address at menarche would most likely be nondifferential with respect to prepregnancy weight and thus would bias results toward the null.

Despite these potential limitations, there are a number of strengths of the current study, including the fact that we have a relatively large sample of African-American women, a group at disproportionate risk of prepregnancy obesity [4] and typically underrepresented in research studies. Although measures of individual-level poverty at menarche and during pregnancy are potential confounders, the difficultly in self-reporting family poverty at menarche (compared to reporting a parent's education level) and the lack of willingness of research participants to report current income prevented accounting for these factors in our models. However, we were able to adjust our models for both individual-level SES at menarche (*grandmother's* education) and at pregnancy (participant's education) and results were similar. To our knowledge, this is the first study to examine neighborhood characteristics at menarche with prepregnancy obesity, which provides a life-course approach linking critical reproductive time points. Future studies are needed to examine such a life-course approach; such studies should capture not only information on the neighborhood at critical points in development, but also individual-level characteristics such as BMI and family income over the life course.

## 5. Conclusions

In summary, we found new evidence that neighborhood-level poverty at menarche is associated with prepregnancy obesity in African-American women. While interventions in adulthood to reduce preconception BMI have shown success [43], intervening earlier in life during adolescence to promote healthy weight throughout a woman's reproductive years may further improve maternal preconception health [44–46]. Including a social perspective in such interventions, such as the impact of neighborhood-level poverty during adolescence, may be promising [47]. This may be especially important for African-American adolescents, who are at highest risk for living in poverty during childhood [48]. Future studies, however, are needed to confirm our findings and to better understand the role of exposures at menarche in health before and during pregnancy.

## Figures and Tables

**Table 1:** Participant characteristics by prepregnancy BMI category (*N* = 144); data are mean ± standard deviation or *n* (%).

Characteristic	Under/normal weight	Overweight	Obese	*P*
*n*	50 (34.7%)	35 (24.3%)	59 (41.0%)	
Age at menarche (years)	12.7 ± 1.6	12.2 ± 2.0	12.0 ± 1.7	0.111
Time since menarche (years)	10.9 ± 5.0	13.9 ± 5.9	17.4 ± 5.5	<0.001
Age at study visit (years)	23.6 ± 4.7	26.1 ± 5.8	29.4 ± 5.6	<0.001
Married/living as married	8 (16.0%)	8 (22.9%)	23 (39.0%)	0.022
Maternal education (years)	12.6 ± 1.4	13.3 ± 1.9	13.0 ± 1.7	0.128
Parity	0.4 ± 0.6	0.9 ± 1.2	1.3 ± 1.2	<0.001
Nulliparous	32 (64.0%)	19 (54.3%)	17 (28.8%)	0.001
Grandmother's education (years)^a^	13.3 ± 2.0	12.7 ± 2.4	13.2 ± 2.1	0.700
Neighborhood-level poverty at menarche (%)	24 ± 13	26 ± 14	30 ± 13	0.054
Quartiles of neighborhood-level poverty at menarche^b^				0.320
Quartile 1	16 (32.0%)	9 (26.5%)	10 (17.0%)	
Quartile 2	12 (24.0%)	11 (32.4%)	13 (22.0%)	
Quartile 3	13 (26.0%)	7 (20.6%)	16 (27.1%)	
Quartile 4	9 (18.0%)	7 (20.6%)	20 (33.9%)	
Current neighborhood-level poverty (%)	27 ± 14	25 ± 15	31 ± 15	0.170
Quartiles of current neighborhood-level poverty^c^				0.639
Quartile 1	12 (24.0%)	11 (32.4%)	12 (20.7%)	
Quartile 2	16 (32.0%)	8 (23.5%)	12 (20.7%)	
Quartile 3	12 (24.0%)	7 (20.6%)	18 (31.0%)	
Quartile 4	10 (20.0%)	8 (23.5%)	16 (27.6%)	

^a^8 women missing grandmother's education.

^b^Quartile 1: 3.4–14%; Quartile 2: ≥14–29%; Quartile 3: ≥29–38%; Quartile 4: ≥38%.

^c^Quartile 1: 0.8–14%; Quartile 2: ≥14–29%; Quartile 3: ≥29–40%; Quartile 4: ≥40%.

**Table 2:** Association of neighborhood-level poverty (in quartiles) at menarche with body mass index category.

Neighborhood-level poverty in quartiles at menarche	Model I		Model II		Model III	
	OR [95% CI OR]	*P*	OR [95% CI OR]	*P*	OR [95% CI OR]	*P*
4 versus 1	2.9 [1.2, 6.9]	0.049	2.6 [1.0, 6.7]	0.048	2.3 [0.9, 6.3]	0.091
3 versus 1	1.7 [0.7, 4.1]	0.870	2.5 [1.0, 6.7]	0.057	1.9 [0.7, 5.2]	0.214
2 versus 1	1.5 [0.6, 3.6]	0.752	2.5 [1.0, 6.6]	0.054	2.3 [0.9, 6.0]	0.101

Model I is unadjusted, Model II is adjusted for maternal age, marital status, and maternal education, and Model III is additionally adjusted for parity.

OR, odds ratio; CI, confidence interval.
